# The Primary Origin of Excellent Dielectric Properties of (Co, Nb) Co-Doped TiO_2_ Ceramics: Electron-Pinned Defect Dipoles vs. Internal Barrier Layer Capacitor Effect

**DOI:** 10.3390/molecules26113230

**Published:** 2021-05-27

**Authors:** Theeranuch Nachaithong, Narong Chanlek, Pairot Moontragoon, Prasit Thongbai

**Affiliations:** 1 Materials Science and Nanotechnology Program, Faculty of Science, Khon Kaen University, Khon Kaen 40002, Thailand; theeranuch_tn@kkumail.com (T.N.); mpairo@kku.ac.th (P.M.); 2 Giant Dielectric and Computational Design Research Group (GD–CDR), Department of Physics, Faculty of Science, Khon Kaen University, Khon Kaen 40002, Thailand; 3 Synchrotron Light Research Institute (Public Organization), 111 University Avenue, Muang District, Nakhon Ratchasima 30000, Thailand; Narong@slri.or.th

**Keywords:** TiO_2_, DFT, SBLC, giant/colossal dielectric permittivity, loss tangent

## Abstract

(Co, Nb) co-doped rutile TiO_2_ (CoNTO) nanoparticles with low dopant concentrations were prepared using a wet chemistry method. A pure rutile TiO_2_ phase with a dense microstructure and homogeneous dispersion of the dopants was obtained. By co-doping rutile TiO_2_ with 0.5 at.% (Co, Nb), a very high dielectric permittivity of ε′ ≈ 36,105 and a low loss tangent of tanδ ≈ 0.04 were achieved. The sample–electrode contact and resistive outer-surface layer (surface barrier layer capacitor) have a significant impact on the dielectric response in the CoNTO ceramics. The density functional theory calculation shows that the 2Co atoms are located near the oxygen vacancy, creating a triangle-shaped 2CoV_o_Ti complex defect. On the other hand, the substitution of TiO_2_ with Nb atoms can form a diamond-shaped 2Nb2Ti complex defect. These two types of complex defects are far away from each other. Therefore, the electron-pinned defect dipoles cannot be considered the primary origins of the dielectric response in the CoNTO ceramics. Impedance spectroscopy shows that the CoNTO ceramics are electrically heterogeneous, comprised of insulating and semiconducting regions. Thus, the dielectric properties of the CoNTO ceramics are attributed to the interfacial polarization at the internal insulating layers with very high resistivity, giving rise to a low loss tangent.

## 1. Introduction

The intensive investigation of a novel giant dielectric oxide (GDO) showing a high dielectric constant (ε′) has fueled research in the field of dielectric materials [1,2,3,4,5,6]. The loss tangent (tanδ) is also an essential factor for capacitor applications. ACu_3_Ti_4_O_12_ (A = Ca, Cd, etc.), the famous GDO, is a widely researched GDO because it can show a large ε′ of ~10^4^ over a wide temperature range [3,7,8,9,10]. This unusual behavior is owing to the existence of a potential barrier at the grain boundaries (GBs); that is, the Schottky barrier. ACu_3_Ti_4_O_12_ ceramics have an electrically heterogeneous microstructure consisting of low conductivity and high conductivity parts. The microstructure is usually analyzed by the brickwork layer model of an internal barrier layer capacitor (IBLC) structure. Therefore, the dielectric properties of many GDOs can be improved by enhancing the electrical properties of the grains and GBs. Although ACu_3_Ti_4_O_12_ ceramics can exhibit a large ε′ response, their tanδ values are very large, which cannot be used in ceramic capacitors [11,12,13,14,15,16,17]. The low-frequency tanδ of ACu_3_Ti_4_O_12_ and other GDOs can be decreased by increasing the resistivity at the GBs (*R*_gb_) via doping ions or designing a small grain-sized microstructure [8,18,19,20].

Recently, a new promising GDO was reported. By co-doping TiO_2_ with aliovalent ions into the rutile structure (i.e., Ti_1−*x*_(Nb_0.5_In_0.5_)*_x_*O_2_), a very high *ε′* > 10^4^ with low tan*δ* ≈ 0.02 can be obtained [21]. The giant dielectric properties, with a low-temperature coefficient of the *ε′* from 80 to 450 K of the Ti_1−*x*_(Nb_0.5_In_0.5_)*_x_*O_2_ ceramics, are much better than those of the ACu_3_Ti_4_O_12_ [15,22,23]. The existence of electron-pinned defect dipoles (EPDD) was suggested to be the primary cause of the observed dielectric properties. In this EPDD model, the diamond-shaped (A = Ti^3^^+^/In^3^^+^/Ti^4^^+^) and triangular-shaped defects were predicted to be closely correlated, resulting in a low tan*δ* and high *ε′* with temperature stability. The former and latter defects can be produced by co-doping with acceptor dopant (e.g., Ga^3+^or In^3^^+^) and donor dopant (e.g., Nb^5^^+^ or Ta^5^^+^) [21,24,25].

Besides the quasi-intrinsic effect of the complex defect dipoles [21,26], extrinsic effects based on interfacial polarization have been widely proposed, such as the surface barrier layer capacitor (SBLC) [27] and IBLC models [28,29]. Although the non-Ohmic contact at the sample–electrode interface (i.e., sample–electrode, SE effect) can have a remarkable impact on the dielectric properties [27], a significant increase in *ε′* is usually accompanied by an enormous tanδ value [24,30]. According to the SBLC and IBLC effects, the insulating layers in co-doped TiO_2_ polycrystalline ceramics can be formed by doping with an acceptor dopant. At the same time, the semiconducting part can be produced by doping with a donor dopant [29,31]. Thus, the origin of the giant dielectric response in co-doped TiO_2_ is still under discussion.

Until recently, many co-doped TiO_2_ systems have been intensively investigated to develop a new co-doped TiO_2_ system that can exhibit a high *ε′* > 10^4^ with low tan*δ*, such as (Ga, Nb), (Ag, Nb), (In, Nb), (Sc, Nb), (In, Ta), and (Y, Nb) co-doped TiO_2_ systems [4,25,31,32,33,34]. To the best of our knowledge, the preparation, characterization, dielectric properties, and formation of defect dipoles of (Co, Nb) co-doped TiO_2_ (CoNTO) ceramics have never been reported. The aims of this study were to prepare and characterize the CoNTO ceramics using a wet chemistry method for obtaining a new co-doped TiO_2_ system that can exhibit a high *ε′* and low tan*δ,* and to clearly explain the primary cause of the dielectric properties.

In the present study, the synthesized CoNTO ceramics with low levels of co-doping concentrations were systematically studied to obtain a high *ε′* and low tan*δ,* and to clarify the primary contribution of the giant dielectric properties in CoNTO ceramics. X-ray photoelectron spectroscopy (XPS), scanning electron microscope (SEM), Raman scattering spectroscopy, and X-ray diffraction (XRD) techniques were used for the characterization of the sintered CoNTO ceramics. Impedance spectroscopy and first-principle calculations were used to evaluate the possible origin of the dielectric response in the CoNTO ceramics. The intrinsic and extrinsic effects were discussed in detail.

## 2. Results and Discussion

**Figure 1:**
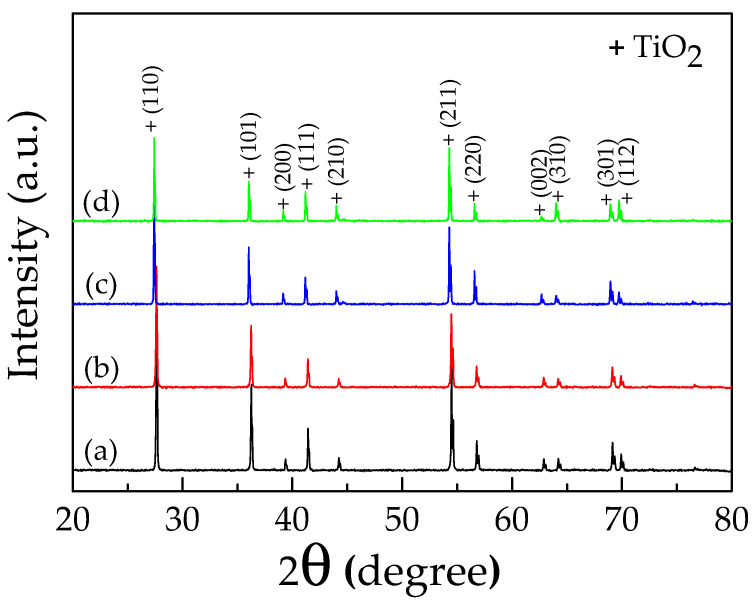
XRD patterns of: (**a**) 0.5% CoNTO; (**b**) 1% CoNTO powders and sintered ceramics; (**c**) 0.5% CoNTO; and (**d**) 1% CoNTO ceramics.

Figure 1 shows the XRD patterns of the 0.5% CoNTO and 1% CoNTO powders and sintered 0.5% CoNTO and 1% CoNTO ceramics. A main phase of the rutile TiO_2_ (JCPDS 21-1276) with a tetragonal structure is clearly observed in all samples with no impurity phase. The lattice parameters (*a* and *c* values) of all the samples calculated by a Rietveld refinement method are summarized in Table 1. Both values are nearly the same as the *a* (4.593 Å) and *c* (2.959 Å) values for the rutile TiO_2_ structure. These calculated values are comparable to those reported in the (In, Nb) co-doped TiO_2_ ceramics 21. The lattice parameters of the 0.5% CoNTO and 1% CoNTO powders are slightly changed. This may be due to the fact that, after the calcination process, the (Co, Nb) dopants did not, or only faintly, substitute into the rutile TiO_2_ structure. The lattice parameters of sintered ceramics had slightly decreased compared to those of the powders with the same doping content. This result may be due to the substitution of rutile TiO_2_ with (Co, Nb) dopants into the structure. On the other hand, the observed increase in the lattice parameters of the 1% CoNTO ceramic compared to those of the 0.5% CoNTO ceramic may be associated with the different ionic radii between the dopants and the host Ti^4+^ ions.

**Figure 2:**
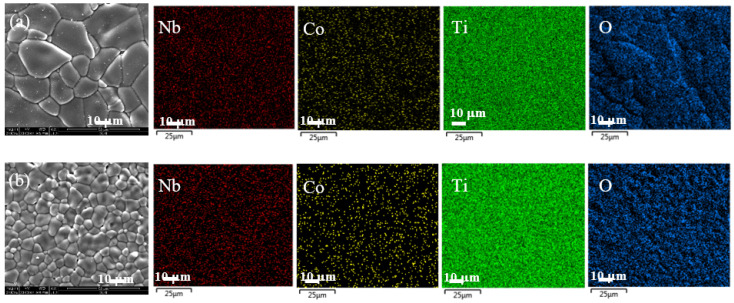
Field emission scanning electron microscope (FE-SEM) images and mapping images of all elements of (**a**) 0.5% CoNTO and (**b**) 1% CoNTO ceramics.

Figure 2a,b show the surface morphologies and the elemental mapping in the 0.5% CoNTO and 1% CoNTO ceramics, respectively. It was found that the dopants were homogeneously dispersed in the microstructure. A dense microstructure consisting of grains and grain boundaries is observed. The average grain size of the 0.5% CoNTO (~16.5 μm) and 1% CoNTO (~7.4 μm) ceramics was significantly changed by variations in the co-doping concentrations. The decrease in the mean grain size of the 1% CoNTO ceramic was likely due to the solute drag mechanism associated with the formation of a space charge at the grain boundaries [32,35,36]. The relative densities of the 0.5% CoNTO and 1% CoNTO ceramics were 94.83% and 97.64%, respectively. Elemental mapping images show homogeneous dispersion of the (Co, Nb) dopants and the major elements of Ti and O throughout the microstructure, without segregation of the dopants at any specific region. 

**Figure 3:**
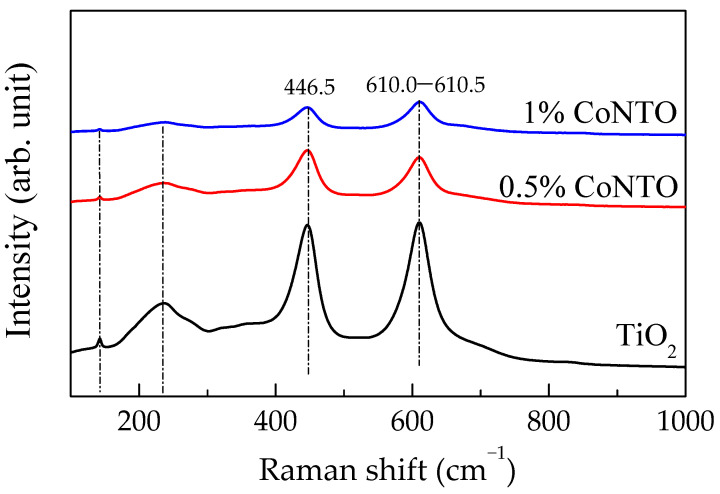
Raman spectra of TiO_2_, 0.5% CoNTO, and 1% CoNTO ceramics.

Figure 3 shows Raman spectra of the 0.5% CoNTO and 1% CoNTO ceramics compared to the undoped TiO_2_ ceramic. The peak positions of the Raman shift of the E_g_ mode were the same position at 445.6 cm^−1^. The peak positions of the A_1g_ mode of the undoped TiO_2_, 0.5% CoNTO, and 1% CoNTO ceramics were 610.5, 610.0, and 610.0 cm^−1^, respectively. Generally, the E_g_ mode in the Raman spectrum of the rutile TiO_2_ is attributed to the vibration mode of oxygen along the *c* axis, which can be correlated with the presence of oxygen vacancies in the structure. The A_1g_ model is related to the vibration of the Ti–O bond [24,28]. The unchanged Raman shift of the E_g_ mode and the A_1g_ model mode may be due to a small doping concentration.

**Figure 4:**
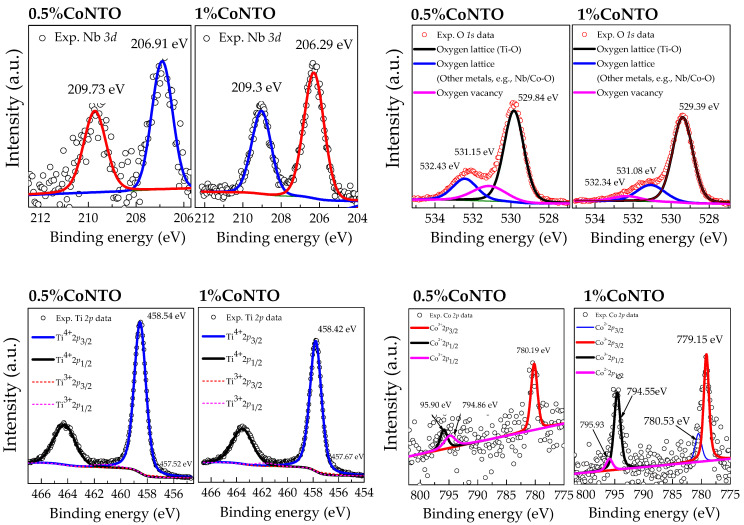
XPS spectra of 0.5% CoNTO and 1% CoNTO ceramics.

The 0.5% CoNTO and 1% CoNTO ceramics were characterized using XPS techniques to clarify the influences of (Co, Nb) co-dopants on the formation of defects in the rutile TiO_2_ structure. XPS spectra of the 0.5% CoNTO and 1% CoNTO ceramics are shown in Figure 4. Two peaks of Nb 3*d* electrons were detected, corresponding to 3*d*_3/2_ and 3*d*_5/2_, respectively, which is entirely consistent with those observed in Nb^5+^ single-doped TiO_2_ materials [21]. Generally, doping a rutile TiO_2_ ceramic with Nb^5+^ can produce free electrons and eventually cause a reduction in Ti^4+^ to Ti^3+^, following the relationship [21]:

(1) $${2\mathrm{TiO}}_{2}{+\mathrm{Nb}}_{2}O_{5}\;\overset{{4\mathrm{TiO}}_{2}}{\to }{\;2\mathrm{Ti}}_{\mathrm{Ti}}{+2\mathrm{Nb}}_{\mathrm{Ti}}^{\cdot }{+8O}_{O}+\frac{1}{2}O_{2},$$

Ti^4+^ + e^−^ → Ti^3+^ (2)

As shown in Figure 4, the binding energies of Ti^3+^ and Ti^4+^ for the 0.5% CoNTO ceramic were about 457.52 and 458.54, respectively, while the binding energies of Ti^3+^ and Ti^4+^ for the 1% CoNTO ceramic were about 457.67 and 458.42 eV, respectively. The oxidation states of Co were Co^2+^ and Co^3+^. As shown in Figure 4 for the XPS spectra of Co2*p*, by fitting the experimental results, the prominent peaks of Co 2*p*_1/2_ and Co 2*p*_3/2_ were observed at about 779.15–780.19 eV and 794.55–794.86 eV, respectively, confirming the existence of the Co^3+^. Additionally, a small peak at relatively higher binding energies was observed. This can be ascribed to the Co^2+^. The XPS spectrum of O1*s* profiles was measured. Three peaks were obtained from the fitted data. The XPS peaks at 529.84, 532.43, and 531.15 eV were ascribed to the oxygen lattice in the bulk ceramic, oxygen lattices of other cation–oxygen bonds, and oxygen vacancies, respectively [21,37]. The substitution of TiO_2_ with acceptor Co^2+^ dopant can result in the existence of oxygen vacancies due to charge compensation, following the relation:

(3) $$\mathrm{CoO}\overset{\;{\mathrm{TiO}}_{2}\;}{\to }{\mathrm{Co}}_{\mathrm{Ti}}^{\prime \prime }{+V}_{O}^{\cdot \cdot }{+O}_{O},$$

**Figure 5:**
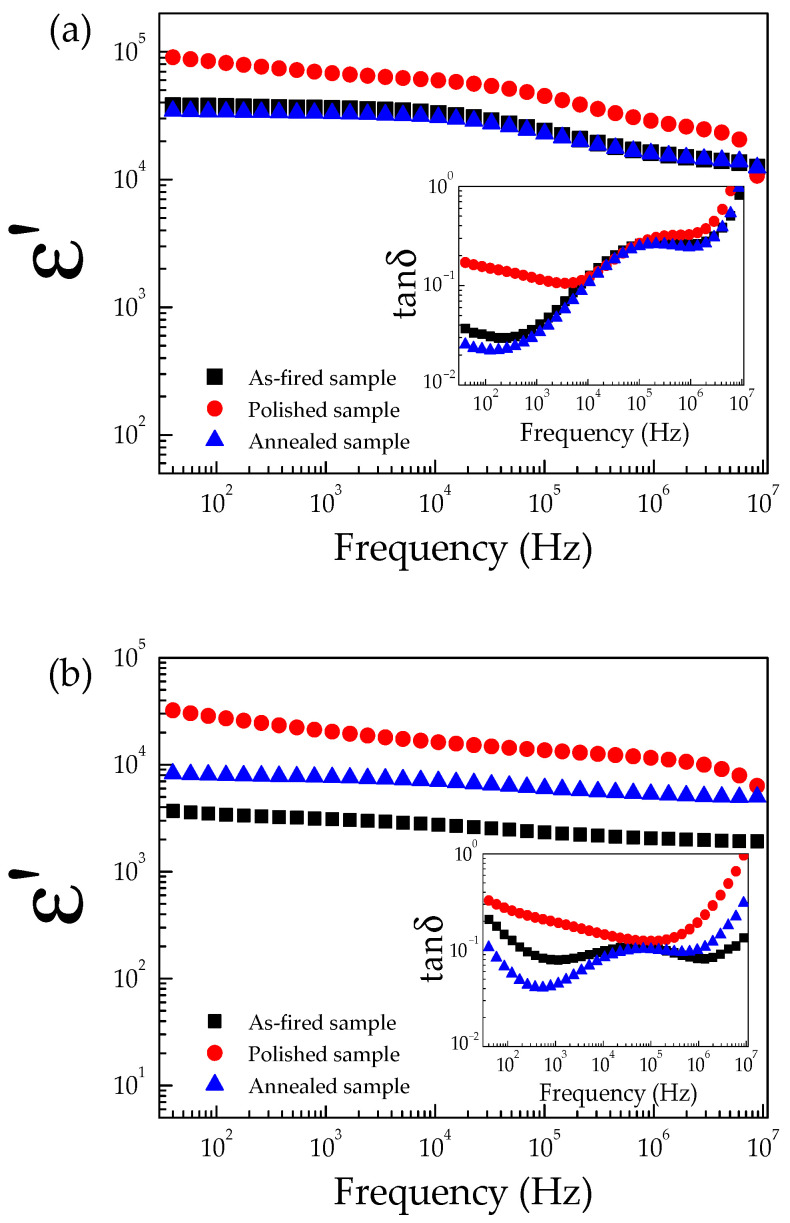
Frequency dependence of ε′ for (**a**) 0.5% CoNTO and (**b**) 1% CoNTO ceramics.

The dielectric properties as a function of frequency at room temperature for the 0.5% CoNTO and 1% CoNTO ceramics are illustrated in Figure 5a,b and its insets. The as-fired samples exhibited ultra-high ε′ values of ~10^3^–10^4^, over the frequency range of 40–10^7^ Hz. At 1 kHz, the ε′ values of the 0.5% CoNTO and 1% CoNTO ceramics were 3.6 × 10^4^ and 3.6 × 10^3^, respectively, while the tanδ values were 0.039 and 0.079, respectively. 

After dielectric properties of the as-fired samples were measured at different temperatures, both sides of the initial electrodes and the outer surface layers of the pellet samples were removed by polishing them with SiC paper (referred to as the polished sample). In this step, the sample thickness was reduced by ~0.12 mm. The polished samples were painted by Ag paste and heated in air at 600 °C for 0.5 h. After that, the dielectric properties of the polished samples were remeasured. It was found that the ε′ of the polished samples significantly increased compared to that of the as-fired samples, especially in a low-frequency range. Furthermore, it was found that the tanδ values of the polished samples significantly increased in frequencies below 10^4^ Hz, as shown in the insets of Figure 5a,b. These results indicated that the outer surface layers of the 0.5% CoNTO and 1% CoNTO ceramics are the insulating layers. Generally, a low-frequency tanδ of many giant dielectric oxides is associated with the DC conduction of the long-range motion of free charge carriers. Removing the resistive outer surface layer results in an increase in conductivity, leading to the observed increase in a low-frequency tanδ. The increased ε′ values of the polished 0.5% CoNTO and 1% CoNTO ceramics were attributed to the non-Ohmic sample–electrode contact.

After the dielectric properties of both polished samples were measured, the electrodes and outer surface layer were removed. In this step, the sample thickness was reduced by ~0.15 mm. Then, the samples were annealed at 1200 °C in the air for 30 min (referred to as the annealed samples). Next, the annealed samples were painted with Ag paste and heated in the air at 600 °C for 0.5 h. After that, the dielectric properties of the annealed samples were measured again. As illustrated in Figure 5a,b, the ε′ and tanδ values of the annealed 0.5% CoNTO sample were recovered to their initial values (as-fired sample), while the ε′ and tanδ values of the annealed 1% CoNTO sample were significantly reduced compared to those of the polished 1% CoNTO sample. At 1 kHz, the ε′ values of the annealed 0.5% CoNTO and 1% CoNTO ceramics were 3.3 × 10^4^ and 7.6 × 10^3^, respectively, while the tanδ values were 0.032 and 0.043, respectively. These results indicated the important role of the insulative outer surface layer that contributed to the dielectric properties of the 0.5% CoNTO and 1% CoNTO ceramics. During the annealing process at 1200 °C, the surfaces of the polished samples were re-oxidized by filling oxygen vacancies on the surface and along the GBs. Free charges on the surfaces and the GB regions were reduced by filling oxygen vacancies with oxygen ions during the annealing process [27,32,38]. Therefore, the SBLC and IBLC mechanisms can be used to explain the dielectric properties of the (Co, Nb) co-doped TiO_2_ ceramics. Thus, it is clearly shown that the SBLC and IBLC effects have a significant impact on the dielectric properties of the 0.5% CoNTO and 1% CoNTO ceramics.

**Figure 6:**
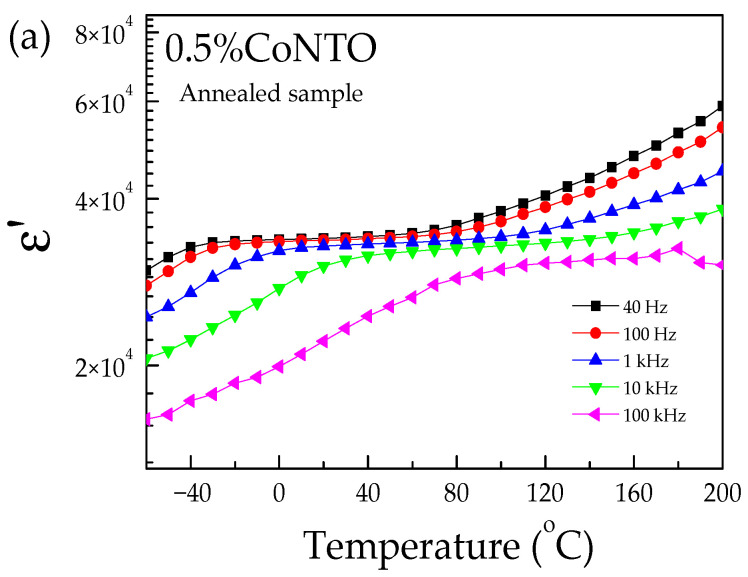

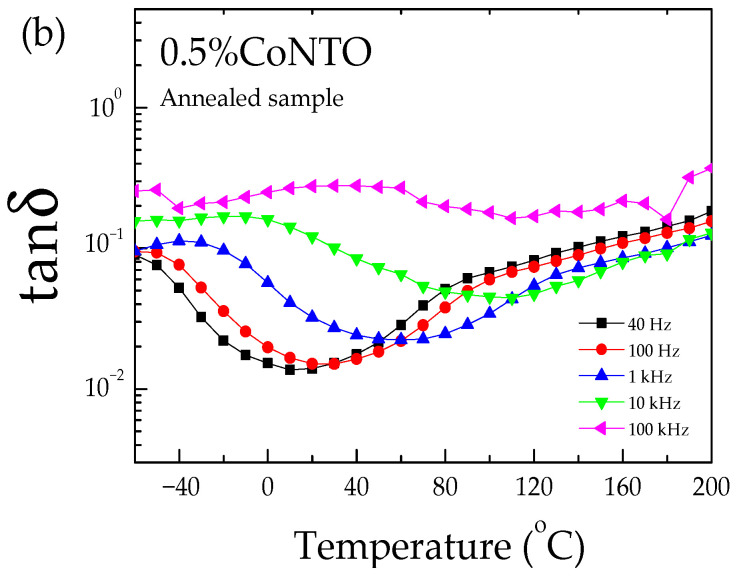
Temperature dependences of (**a**) ε′ and (**b**) tanδ of 0.5% CoNTO ceramic.

**Figure 7:**
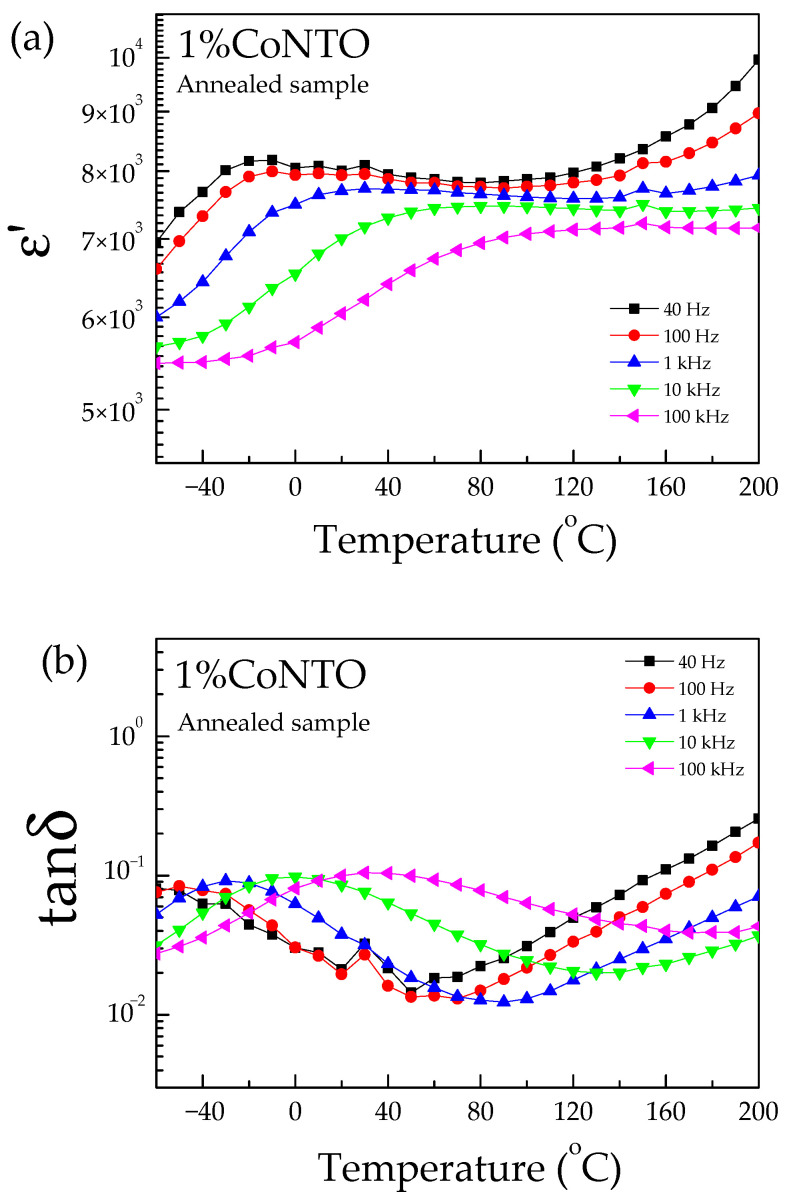
Temperature dependences of (**a**) ε′ and (**b**) tanδ of 1% CoNTO ceramic.

Figure 6 and Figure 7 show the temperature dependences of the ε′ and tanδ values of the 0.5% CoNTO and 1% CoNTO ceramics. The relaxation peak of tanδ was observed in the 0.5% CoNTO and 1% CoNTO ceramics in the temperature range from −60 to 40 °C. The dielectric relaxation was likely attributed to the electrical response of internal insulating interfaces between grains [39]. The dielectric relaxation in a low temperature range is similar to that observed in ACu_3_Ti_4_O_12_ ceramics. This result may be due to the Maxwell–Wagner polarization relaxation at the insulating GBs [40,41]. The relaxation peak of tanδ shifts to high temperatures as the frequency increases, indicating a thermally activated relaxation mechanism. 

**Figure 8:**
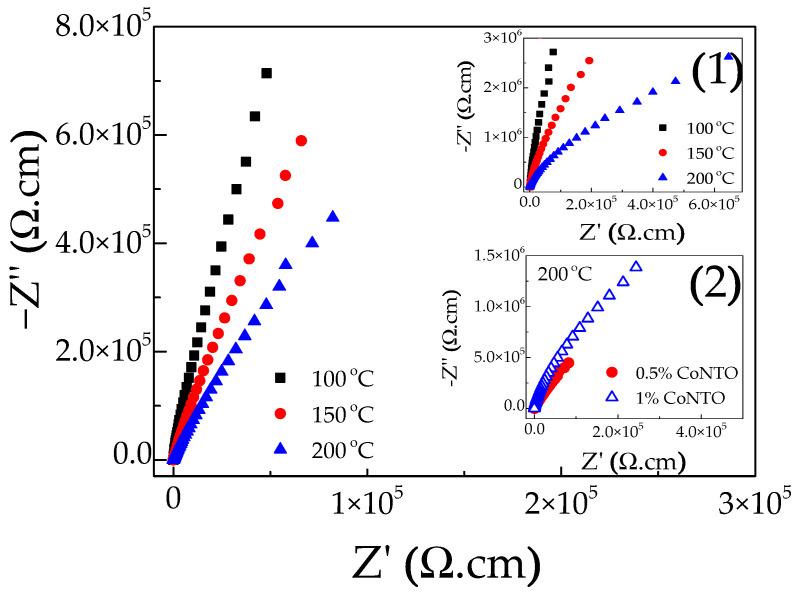
Impedance spectra at different temperatures (100–200 °C) for 0.5% CoNTO ceramic. Inset (**1**) shows impedance spectra of 1% CoNTO ceramic at different temperatures and inset (**2**) shows the comparison of impedance spectra at 200 °C for 0.5% CoNTO and 1% CoNTO ceramics.

By using impedance spectroscopy analysis, the electrical properties of the grains and GBs can be characterized [8]. As shown in Figure 8 and the inset (1), only parts of large arcs are observed in a high-temperature range (100–200 °C) for the 0.5% CoNTO and 1% CoNTO ceramics, indicating the electrical response of the insulating parts (GBs and resistive outer surface layer). The nonzero intercept of the impedance spectra on the Z′ axis was observed for all the ceramics (not shown), confirming the existence of semiconducting grains with the grain resistance of R_g_ ~40 and 25 Ω.cm for the 0.5% CoNTO and 1% CoNTO ceramics, respectively. Therefore, the 0.5% CoNTO and 1% CoNTO ceramics are electrically heterogeneous and comprised of insulating and semiconducting parts. Furthermore, the diameter of the large arc decreased with increasing temperature, indicating the decrease in total resistance of the insulating parts (R_i_). Thus, the colossal permittivity in the 0.5% CoNTO and 1% CoNTO ceramics prepared by a wet chemical process method may be primarily caused by extrinsic factors resulting from the IBLC and SBLC effects. The result indicates that the dielectric properties are associated with the electrical responses of grain and grain boundaries. As shown in the inset (2) of Figure 8, although the arc of the 0.5% CoNTO and 1% CoNTO ceramics cannot be seen at 200 °C, it is clearly observed that the arc of the 1% CoNTO ceramic was much larger than that of the 0.5% CoNTO ceramic. This result indicates that the R_i_ of the 1% CoNTO ceramic was larger than that of the 0.5% CoNTO ceramic. As shown in Figure 2a,b, the mean grain size of the 1% CoNTO ceramic was smaller than that of the 0.5% CoNTO ceramic. Thus, the number of insulating GBs per volume (GB density) of the 1% CoNTO ceramic should be higher than that of the 0.5% CoNTO ceramic, resulting in the increased R_i_.

**Figure 9:**
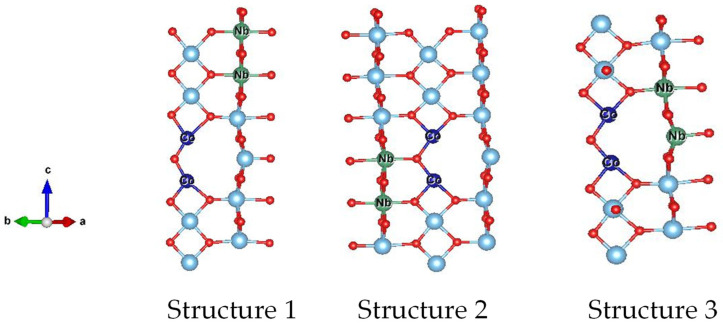
Energy-preferable structure of the 2CoV_o_ triangular defect and 2Nb diamond with different configurations.

To clarify the possible origin of the colossal dielectric properties in 0.5% CoNTO and 1% CoNTO ceramics, we calculated the most stable configurations of 2CoV_o_TiO_2_ and 2NbTiO_2_. To investigate the lowest energy configuration of the 0.5% CoNTO and 1% CoNTO ceramics, the 2CoV_o_ triangular and 2Nb diamond defects were placed into the TiO_2_ structure simultaneously in three configurations, for structures 1–3, as shown in Figure 9. The stable structure of 2NbTiO_2_ is structure 1 due to the lowest total energy. This result indicates that the 2CoV_o_ triangular defect does not prefer to be close to the 2Nb diamond defect. For the formation of defect clusters of EPDDs, these two types of defects must be close together. However, according to DFT calculations, the lowest total energy can be obtained when the 2CoV_o_ triangular and 2Nb diamond defects are far away. Therefore, it is reasonable to suggest that the colossal dielectric properties of the 0.5% CoNTO and 1% CoNTO ceramics were attributed to the SBLC and IBLC effects, in which the electron hopping mechanism between Ti^3+^ and Ti^4+^ ions occurred inside the semiconducting grains of the 0.5% CoNTO and 1% CoNTO ceramics.

## 3. Materials and Methods

(Nb_2/3_Co_1/3_)*_x_*Ti_1−*x*_O_2_ (CoNTO) powders with *x* = 0.5% (0.5% CoNTO) and 1% (1% CoNTO) were prepared by a wet chemistry method. Co(NO_3_)_2_•6H_2_O (Kanto chemical, >99.5%), Diisopropoxytitanium bis(acetylacetonate) (C_16_H_28_O_6_Ti, Sigma–Aldrich), NbCl_5_ (Sigma–Aldrich, >99.9%), deionized water, and citric acid were used as the starting raw materials. First, Co(NO_3_)_2_•6H_2_O and NbCl_5_ were dissolved in an aqueous solution of citric acid under constant stirring at ~25 °C (solution A). Second, a C_16_H_28_O_6_Ti solution was dropped into solution A at 130 °C until a viscous gel was obtained. Third, a viscous gel was heated at 350 °C in an oven for 1 h to form dried porous precursors. Then, the resulting dried precursors were ground and calcined in air at 1000 °C for 12 h to produce the rutile phase in the 0.5% CoNTO and 1% CoNTO powders. Next, the obtained 0.5% CoNTO and 1% CoNTO powders were carefully ground. After that, the powders were pressed into pellets of ~1.0 mm in thickness and ~9.5 mm in diameter. Finally, the pellets were sintered at 1450 °C for 5 h. The heating and cooling rates were 2 °C/min and ~10 °C/min, respectively.

The prepared powders and sintered ceramics were characterized using XRD (PANalytical, EMPYREAN), SEM (FEI, QUANTA 450), XPS (AXIS Ultra DLD, UK), and Raman (Horiba Jobin-Yvon T64000) techniques. The densities of the sintered samples were measured using an Archimedes’ method. For the measurement of the dielectric properties, the top and bottom surfaces of the sintered samples with thickness < 1 mm were painted with Ag paste and heated in air at 600 °C for 0.5 h to make good electrode contact. The dielectric properties of the as-fired 0.5% CoNTO and 1% CoNTO ceramics were tested using a KEYSIGHT E4990A impedance analyzer over the frequency range from 40 to 10^7^ Hz using an oscillation voltage of 0.5 V. The dielectric properties as a function of temperature were measured using a step increase of 10 °C from −60 to 200 °C.

The stable configuration of the (Co, Nb) co-doped rutile TiO_2_ was investigated using the density functional theory (DFT) implemented in the Vienna ab initio simulation package (VASP). According to the pseudopotential used in this work, the projector augments wave approach and the Perdew–Burke–Ernzerhof (PBE) form of exchange–correlation functional was chosen. The 600-eV plane-wave energy cutoff and 3 × 3 × 3 k-point samplings with Monkhorst–Pack scheme were successfully tested. The conjugate–gradient algorithm was carried out, and the force acting on each ion was calculated by the Hellmann–Feynman theorem.

## 4. Conclusions

(Nb_0.67_Co_0.33_)*_x_*Ti_1−*x*_O_2_ ceramics with different co-dopant concentrations were successfully prepared using a wet chemistry method. A highly dense microstructure was obtained in all ceramics. High ε′ ≈ 10^3^–10^4^ and very low tanδ ≈ 0.032–0.079 at 1 kHz were achieved. The XPS analysis indicated that the substitution of TiO_2_ with Co^2+^ and/or Co^3+^ caused the existence of $V_{O}^{\cdot \cdot }$ for charge compensation, while doping TiO_2_ with Nb^5+^ could cause the existence of free electrons, giving rise to the electron hopping mechanism between Ti^4+^ and Ti^3+^ in the semiconducting grains. Examination of the possible formation of defect structures was performed using a DFT calculation. The 2Nb diamond did not correlate with the 2CoV_o_ triangular shapes, indicating that there was no EPDD. According to the impedance spectroscopy and DFT calculation, it can reasonably be suggested that the origins of the colossal dielectric properties are attributable to the IBLC and SBLC effects.

## Figures and Tables

**Table 1:** Lattice parameters of (Co_0.33_Nb_0.67_)*_x_*Ti_1-*x*_O_2_ powders and ceramics.

Sample	Lattice Parameter (Å)	
	*a*	*c*
0.5% CoNTO powder	4.596	2.962
1% CoNTO powder	4.596	2.962
0.5% CoNTO ceramic	4.593	2.960
1% CoNTO ceramic	4.595	2.961

## Data Availability

The data presented in this study are available in article.

## References

[1] Xu Z., Li L., Wang W., Lu T. (2019). Colossal permittivity and ultralow dielectric loss in (Nd_0.5_Ta_0.5_)xTi_1-x_O_2_ ceramics. Ceram. Int..

[2] Peng Z., Wang J., Liang P., Zhu J., Zhou X., Chao X., Yang Z. (2020). A new perovskite-related ceramic with colossal permittivity and low dielectric loss. J. Eur. Ceram. Soc..

[3] Peng Z., Liang P., Wang J., Zhou X., Zhu J., Chao X., Yang Z. (2020). Interfacial effect inducing thermal stability and dielectric response in CdCu_3_Ti_4_O_12_ ceramics. Solid State Ion..

[4] Zhou X., Liang P., Zhu J., Peng Z., Chao X., Yang Z. (2020). Enhanced dielectric performance of (Ag_1/4_Nb_3/4_)_0.01_Ti_0.99_O_2_ ceramic prepared by a wet-chemistry method. Ceram. Int..

[5] Liang P., Zhu J., Wu D., Peng H., Chao X., Yang Z. (2021). Good dielectric performance and broadband dielectric polarization in Ag, Nb co-doped TiO_2_. J. Am. Ceram. Soc..

[6] Zhu J., Wu D., Liang P., Zhou X., Peng Z., Chao X., Yang Z. (2021). Ag^+^/W^6+^ co-doped TiO_2_ ceramic with colossal permittivity and low loss. J. Alloys Compd..

[7] Sinclair D.C., Adams T.B., Morrison F.D., West A.R. (2002). CaCu_3_Ti_4_O_12_: One-step internal barrier layer capacitor. Appl. Phys. Lett..

[8] Adams T., Sinclair D., West A. (2006). Characterization of grain boundary impedances in fine- and coarse-grained CaCu_3_Ti_4_O_12_ ceramics. Phys. Rev. B.

[9] Peng Z., Zhou X., Wang J., Zhu J., Liang P., Chao X., Yang Z. (2020). Origin of colossal permittivity and low dielectric loss in Na_1/3_Cd_1/3_Y_1/3_Cu_3_Ti_4_O_12_ ceramics. Ceram. Int..

[10] Zhao N., Liang P., Wu D., Chao X., Yang Z. (2019). Temperature stability and low dielectric loss of lithium-doped CdCu_3_Ti_4_O_12_ ceramics for X9R capacitor applications. Ceram. Int..

[11] Lunkenheimer P., Fichtl R., Ebbinghaus S., Loidl A. (2004). Nonintrinsic origin of the colossal dielectric constants in CaCu_3_Ti_4_O_12_. Phys. Rev. B.

[12] Li M., Cai G., Zhang D.F., Wang W.Y., Wang W.J., Chen X.L. (2008). Enhanced dielectric responses in Mg-doped CaCu_3_Ti_4_O_12_. J. Appl. Phys..

[13] Ni L., Chen X.M. (2010). Enhancement of Giant Dielectric Response in CaCu_3_Ti_4_O_12_ Ceramics by Zn Substitution. J. Am. Ceram. Soc..

[14] Ni L., Chen X.M., Liu X.Q. (2010). Structure and modified giant dielectric response in CaCu_3_(Ti_1−x_Sn_x_)_4_O_12_ ceramics. Mater. Chem. Phys..

[15] Yang Z., Zhang L., Chao X., Xiong L., Liu J. (2011). High permittivity and low dielectric loss of the Ca_1−x_Sr_x_Cu_3_Ti_4_O_12_ ceramics. J. Alloys Compd..

[16] Sun L., Zhang R., Wang Z., Cao E., Zhang Y., Ju L. (2016). Microstructure and enhanced dielectric response in Mg doped CaCu_3_Ti_4_O_12_ ceramics. J. Alloys Compd..

[17] Jumpatam J., Putasaeng B., Yamwong T., Thongbai P., Maensiri S. (2013). Enhancement of giant dielectric response in Ga-doped CaCu_3_Ti_4_O_12_ ceramics. Ceram. Int..

[18] Thongbai P., Yamwong T., Maensiri S., Amornkitbamrung V., Chindaprasirt P. (2014). Improved Dielectric and Nonlinear Electrical Properties of Fine-Grained CaCu_3_Ti_4_O_12_ Ceramics Prepared by a Glycine-Nitrate Process. J. Am. Ceram. Soc..

[19] Boonlakhorn J., Thongbai P. (2020). Dielectric properties, nonlinear electrical response and microstructural evolution of CaCu_3_Ti_4-x_Sn_x_O_12_ ceramics prepared by a double ball-milling process. Ceram. Int..

[20] Jumpatam J., Putasaeng B., Chanlek N., Boonlakhorn J., Thongbai P., Phromviyo N., Chindaprasirt P. (2021). Significantly improving the giant dielectric properties of CaCu_3_Ti_4_O_12_ ceramics by co-doping with Sr^2+^ and F^-^ ions. Mater. Res. Bull..

[21] Hu W., Liu Y., Withers R.L., Frankcombe T.J., Norén L., Snashall A., Kitchin M., Smith P., Gong B., Chen H. (2013). Electron-pinned defect-dipoles for high-performance colossal permittivity materials. Nat. Mater..

[22] Wu J., Nan C.-W., Lin Y., Deng Y. (2002). Giant Dielectric Permittivity Observed in Li and Ti Doped NiO. Phys. Rev. Lett..

[23] Chouket A., Bidault O., Optasanu V., Cheikhrouhou A., Cheikhrouhou-Koubaa W., Khitouni M. (2016). Enhancement of the dielectric response through Al-substitution in La_1.6_Sr_0.4_NiO_4_ nickelates. RSC Adv..

[24] Hu W., Lau K., Liu Y., Withers R.L., Chen H., Fu L., Gong B., Hutchison W. (2015). Colossal Dielectric Permittivity in (Nb+Al) Codoped Rutile TiO_2_ Ceramics: Compositional Gradient and Local Structure. Chem. Mater..

[25] Nachaithong T., Tuichai W., Kidkhunthod P., Chanlek N., Thongbai P., Maensiri S. (2017). Preparation, characterization, and giant dielectric permittivity of (Y^3+^ and Nb^5+^) co–doped TiO_2_ ceramics. J. Eur. Ceram. Soc..

[26] Han H., Dufour P., Mhin S., Ryu J.H., Tenailleau C., Guillemet-Fritsch S. (2015). Quasi-intrinsic colossal permittivity in Nb and In co-doped rutile TiO_2_ nanoceramics synthesized through a oxalate chemical-solution route combined with spark plasma sintering. Phys. Chem. Chem. Phys..

[27] Nachaithong T., Kidkhunthod P., Thongbai P., Maensiri S. (2017). Surface barrier layer effect in (In + Nb) co-doped TiO_2_ ceramics: An alternative route to design low dielectric loss. J. Am. Ceram. Soc..

[28] Liu G., Fan H., Xu J., Liu Z., Zhao Y. (2016). Colossal permittivity and impedance analysis of niobium and aluminum co-doped TiO_2_ ceramics. RSC Adv..

[29] Wu Y.Q., Zhao X., Zhang J.L., Su W.B., Liu J. (2015). Huge low-frequency dielectric response of (Nb,In)-doped TiO_2_ ceramics. Appl. Phys. Lett..

[30] Nachaithong T., Thongbai P., Maensiri S. (2017). Colossal permittivity in (In_1/2_Nb_1/2_)_x_Ti_1−x_O_2_ ceramics prepared by a glycine nitrate process. J. Eur. Ceram. Soc..

[31] Tuichai W., Danwittayakul S., Chanlek N., Thongbai P., Maensiri S. (2017). High-performance giant-dielectric properties of rutile TiO_2_ co-doped with acceptor-Sc^3+^ and donor-Nb^5+^ ions. J. Alloys Compd..

[32] Tuichai W., Thongyong N., Danwittayakul S., Chanlek N., Srepusharawoot P., Thongbai P., Maensiri S. (2017). Very low dielectric loss and giant dielectric response with excellent temperature stability of Ga^3+^ and Ta^5+^ co-doped rutile-TiO_2_ ceramics. Mater. Des..

[33] Dong W., Hu W., Berlie A., Lau K., Chen H., Withers R.L., Liu Y. (2015). Colossal Dielectric Behavior of Ga+Nb Co-Doped Rutile TiO_2_. ACS Appl. Mater. Interfaces.

[34] Dong W., Hu W., Frankcombe T.J., Chen D., Zhou C., Fu Z., Candido L., Hai G., Chen H., Li Y. (2017). Colossal permittivity with ultralow dielectric loss in In + Ta co-doped rutile TiO_2_. J. Mater. Chem. A.

[35] Rahaman M.N. (2003). Ceramic Processing and Sintering.

[36] Thongbai P., Jumpatam J., Yamwong T., Maensiri S. (2012). Effects of Ta^5+^ doping on microstructure evolution, dielectric properties and electrical response in CaCu_3_Ti_4_O_12_ ceramics. J. Eur. Ceram. Soc..

[37] Cheng X., Li Z., Wu J. (2015). Colossal permittivity in ceramics of TiO_2_Co-doped with niobium and trivalent cation. J. Mater. Chem. A.

[38] Wang C.C., Zhang L.W. (2006). Surface-layer effect in CaCu_3_Ti_4_O_12_. Appl. Phys. Lett..

[39] Tuichai W., Danwittayakul S., Maensiri S., Thongbai P. (2016). Investigation on temperature stability performance of giant permittivity (In + Nb) in co-doped TiO_2_ ceramic: A crucial aspect for practical electronic applications. RSC Adv..

[40] Liu J., Duan C.-G., Yin W.-G., Mei W., Smith R., Hardy J. (2004). Large dielectric constant and Maxwell-Wagner relaxation in Bi_2∕3_Cu_3_Ti_4_O_12_. Phys. Rev. B.

[41] Thongbai P., Jumpatam J., Putasaeng B., Yamwong T., Maensiri S. (2012). The origin of giant dielectric relaxation and electrical responses of grains and grain boundaries of W-doped CaCu_3_Ti_4_O_12_ ceramics. J. Appl. Phys..

